# VrNAC25 Promotes Anthocyanin Synthesis in Mung Bean Sprouts Synergistically with VrMYB90

**DOI:** 10.3390/plants14233667

**Published:** 2025-12-02

**Authors:** Yaolei Zhu, Yao Liu, Fangfang You, Zixin Wan, Meilian Guo, Menghan Lu, Lu Yang, Xuezhu Wang, Jiajun Yang, Li Jia, Nana Su

**Affiliations:** 1 School of Food and Drug, Luoyang Normal University, Luoyang 471934, China; zhuyaolei@lynu.edu.cn (Y.Z.); wanzixin025@163.com (Z.W.); 19987352272@163.com (M.G.); 18736926820@163.com (M.L.); 13461080915@163.com (L.Y.); wangxuezhu09@163.com (X.W.); 18461011873@163.com (J.Y.); 2 School of Ecological Engineering, Henan Forestry Vocational College, Luoyang 471002, China; liuyao502589402@163.com; 3 Luoyang Customs, Luoyang 471000, China; youf1221@126.com; 4 College of Life Sciences, Nanjing Agricultural University, Nanjing 210014, China

**Keywords:** anthocyanins, mung bean, sprout, VrNAC25, VrMYB90

## Abstract

Anthocyanins pigment plant tissues, mitigate biotic and abiotic stresses, and deliver human health benefits; raising their content in mung bean (*Vigna radiata*) sprouts is a long-standing research target. Transcriptome analysis identified VrNAC25, a NAC transcription factor whose expression closely parallels anthocyanin accumulation; functional validation in mung bean confirmed that VrNAC25 acts as a positive regulator of the pathway. Although VrNAC25 does not bind to the promoters of the key structural genes *VrDFR* or *VrLDOX*, it indirectly controls anthocyanin synthesis by interacting with the core R2R3-MYB activator VrMYB90, previously established as the central regulator of anthocyanin production in mung beans. This interaction operates at both transcriptional and protein levels, thereby amplifying the expression of downstream structural genes and boosting pigment accumulation. Our findings refine the molecular network governing anthocyanin biosynthesis in sprouts and provide a clear theoretical basis for breeding or biotechnological strategies aimed at enhancing the nutritional quality and commercial value of mung bean products through light treatment or by selecting an anthocyanin-rich mung bean variety.

## 1. Introduction

Mung beans are rich in nutrients and bioactive compounds, including proteins, vitamin C, trace elements, and flavonoids [1], and exhibit antioxidant, antibacterial, anticancer, and anti-inflammatory activities [2]. Anthocyanins, a subclass of flavonoids widely distributed in plants, are composed of anthocyanidins conjugated with sugar moieties (glycosides) [3]. They are typically stored in vacuoles and impart red, blue, and purple pigmentation to diverse plant organs and tissues [4]. Different from traditional etiolated mung bean sprouts, mung bean sprouts rich in anthocyanins have gained increasing popularity in the market due to their higher nutritional and health value as a kind of natural antioxidants. However, the regulatory mechanism of anthocyanin synthesis in mung bean sprouts still needs to be further analyzed.

The anthocyanin biosynthetic pathway has been well characterized: anthocyanins are synthesized in the cytoplasm through the flavonoid branch of the phenylpropanoid pathway and subsequently transported into vacuoles for storage [5]. Numerous structural genes participate in anthocyanin biosynthesis, including phenylalanine ammonia-lyase (*PAL*), cinnamate 4-hydroxylase (*C4H*), 4-coumarate:CoA ligase (*4CL*), chalcone synthase (*CHS*), chalcone isomerase (*CHI*), flavanone 3-hydroxylase (*F3H*), flavonoid 3′-hydroxylase (*F3′H*), dihydroflavonol 4-reductase (*DFR*), anthocyanidin synthase (*ANS*), and UDP glucose:flavonoid 3-O-glucosyltransferase (*UFGT*) [6]. In addition, several regulatory genes participate in the regulation of anthocyanin biosynthesis. The canonical regulatory mechanism in plants involves the MBW ternary complex, consisting of MYB, bHLH, and WD40 transcription factors [7]. Recent studies have revealed that NAC (NAM/ATAF/CUC) transcription factors also participate in anthocyanin biosynthesis, acting either directly or indirectly [8].

NAC transcription factors form one of the largest families in plants and play essential roles in growth, development, stress responses, and secondary metabolism [9]. A typical NAC protein contains a conserved N-terminal NAC domain and a variable C-terminal transcriptional regulatory region [10]. Studies on NAC-mediated regulation of plant secondary metabolites remain limited, with most focusing on carotenoid and flavonoid biosynthesis. As a type of flavonoid, anthocyanins are also regulated by NAC transcription factors. Previous studies have demonstrated that NAC transcription factors regulate anthocyanin biosynthesis in various plants, including ANAC032 andANAC042 in *Arabidopsis* and MdNAC52 in apple. In *Arabidopsis thaliana*, ANAC032 negatively regulates anthocyanin biosynthesis under stress [11], whereas overexpression of *ANAC078* enhances flavonoid accumulation and upregulates anthocyanin biosynthetic genes [12]. ANAC042 has also been identified as a negative regulator of anthocyanin accumulation [13]. In apple, MdNAC52 activates anthocyanin biosynthetic genes by binding to the promoters of *MdMYB9* and *MdMYB11* [8]. In peach, the bHLH protein PpBL interacts with the NAC-type transcription factor PpNAC1 to activate the promoter of *PpMYB10.1*, thereby promoting anthocyanin accumulation [14].

In our previous study, we identified a core factor VrMYB90 that regulates anthocyanin synthesis in mung bean sprouts [15]. VrMYB90 directly binds to the promoters of key structural genes involved in anthocyanin biosynthesis and activates their transcription, thereby positively regulating anthocyanin accumulation in mung bean sprouts. To further elucidate the molecular regulatory network controlling anthocyanin biosynthesis in mung bean, transcriptomic analyses were performed on two varieties: M0313 (high anthocyanin) and SuLv1 (non-pigmented). A highly expressed transcription factor, VrNAC25, was identified (Figure S1). Subsequent experiments confirmed that VrNAC25 positively regulates anthocyanin biosynthesis in mung bean sprouts, acting indirectly through interaction with VrMYB90. This study unveils the VrNAC25–VrMYB90 module as a novel, positive regulatory node in mung bean anthocyanin synthesis, offering an immediately exploitable gene cassette for marker-assisted breeding of high-antioxidant sprouts. By stacking or fine-tuning these two alleles, breeders can sustainably produce color-rich, health-promoting mung bean products without compromising yield, accelerating the crop’s entry into the functional-food market.

## 2. Results

### 2.1. Characteristics of VrNAC25 Protein

**Figure 1:**
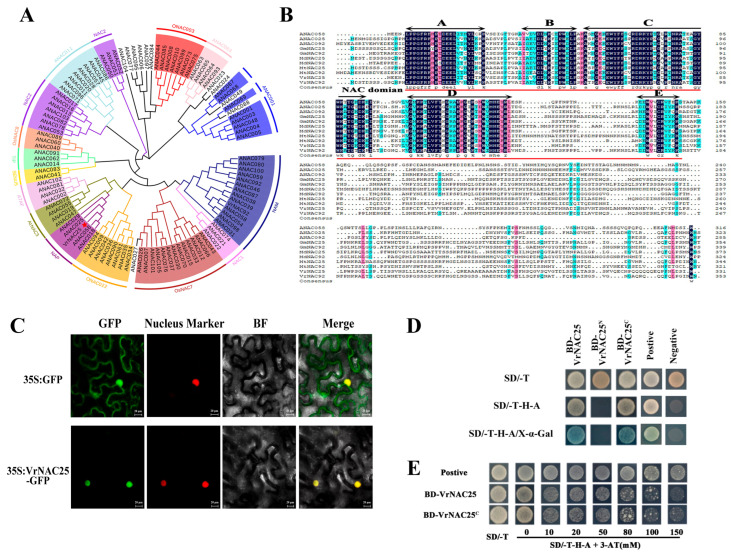
Characteristics of VrNAC25 Protein. (**A**) Phylogenetic tree analysis of VrNAC25 and *Arabidopsis* NAC proteins. (**B**) Amino acid sequence alignment of VrNAC25 proteins. (**C**) Subcellular localization of VrNAC25 proteins in *Nicotiana tabacum* leaves. (**D**) Transcriptional activation activity and transcriptional activation domain analysis of VrNAC25 in yeast. (**E**) Inhibition assay of transcriptional activation activities of VrNAC25 in yeast.

A phylogenetic tree including VrNAC25 and NAC transcription factors from *Arabidopsis thaliana* was constructed using the neighbor-joining method. The results showed that VrNAC25 is most closely related to ANAC025 of *A. thaliana* and clusters into the same branch with ANAC056 and ANAC018 (Figure 1A). Amino-acid sequence alignment revealed that VrNAC25 possesses a typical NAC domain comprising five conserved subdomains (A–E), whereas the C-terminal region is highly divergent (Figure 1B). To investigate the subcellular localization of VrNAC25, *Agrobacterium tumefaciens* strain GV3101 was used to infiltrate tobacco leaves. In *Nicotiana tabacum* leaves, fluorescence from the 35S:VrNAC25-GFP fusion protein was exclusively detected in the nuclei and colocalized with the PIP2A-RFP nuclear marker, whereas no signal was observed in leaves infiltrated with the empty vector (Figure 1C). These results confirm that VrNAC25 is localized in the nucleus and belongs to nuclear protein.

To examine transcriptional activity, full-length VrNAC25 and its N-terminal (1–151 aa) and C-terminal (152–321 aa) fragments were fused to the GAL4 DNA-binding domain (BD) and transformed into AH109 yeast cells. The transformed strains were cultured on SD/-T, SD/-T-H-A deficient media, and SD/-T-H-A media supplemented with X-α-Gal, with negative and positive controls set. Yeast cells transformed with BD-VrNAC25 and BD-VrNAC25C grew normally on both SD/-T and SD/-T-H-A media and turned blue on SD/-T-H-A media supplemented with X-α-Gal. In contrast, yeast cells transformed with BD-VrNAC25^N^ only grew on SD/-T media but failed to grow on SD/-T-H-A media (Figure 1D). In the transcriptional activation inhibition assay, compared with the positive control, yeast cells transformed with BD-VrNAC25 and BD-VrNAC25^C^ showed weak growth on media containing 80 mM 3-AT (Figure 1E). These results indicate that VrNAC25 has strong transcriptional activity, and its transcriptional activation domain is located in the C-terminal region.

### 2.2. VrNAC25 Expression Is Highly Correlated with Anthocyanin Biosynthesis in Mung Bean Sprouts

**Figure 2:**
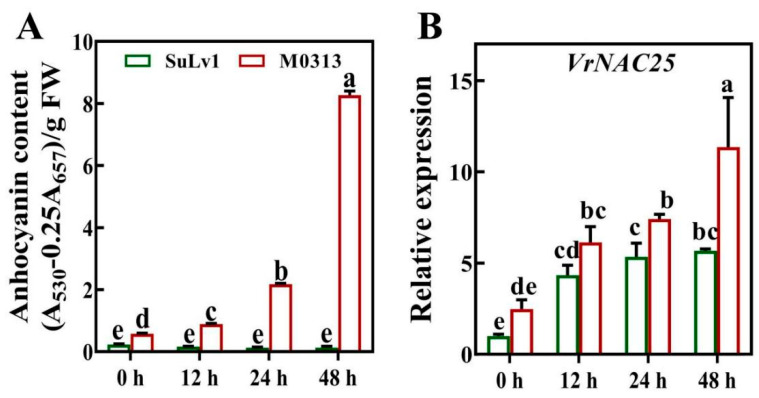
Relative expression levels of *VrNAC25*. (**A**) The content of anthocyanin in hypocotyls of variety SuLv 1 and Mo313 under different light time treatments. (**B**) The relative expression levels of *VrNAC25* transcription factors in hypocotyls of variety SuLv 1 and Mo313 under different light time treatments. Values are means ± standard deviation (SD). Error bars represent the standard deviation (*n* = 3). Statistical significance determined using Student’s *t*-test (*p* < 0.05).

To verify whether anthocyanin biosynthesis is regulated by VrNAC25, relevant experiments were conducted. VrNAC25 expression closely mirrored the pattern of anthocyanin accumulation in hypocotyls (Figure 2). In particular, the expression level of *VrNAC25* in the variety rich in anthocyanins, ‘M0313’, was significantly higher than that in the anthocyanin-lacking variety ‘SuLv1’. Moreover, the expression of *VrNAC25* gradually increased with prolonged light exposure, along with anthocyanin biosynthesis. These results suggested that VrNAC25 might be a regulator of anthocyanin biosynthesis in mung bean sprouts.

### 2.3. VrNAC25 Positively Regulates Anthocyanin Biosynthesis in Mung Bean

**Figure 3:**
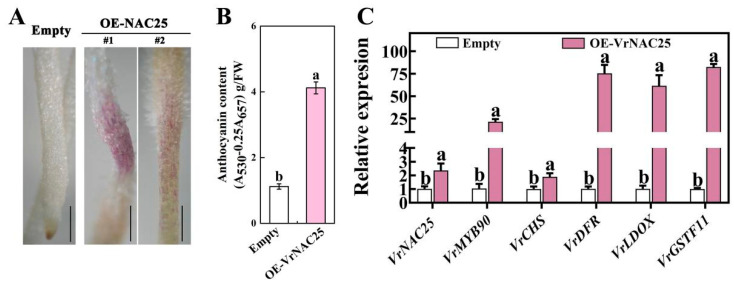
Verification of VrNAC25 functions in mung bean. (**A**) Phenotype of transgenic hairy roots. Two independent transgenic lines, #1 and #2, were represented. Bar = 1 mm; (**B**) contents of anthocyanins in transgenic hairy roots; (**C**) expression levels of *VrNAC25* and anthocyanin biosynthesis-related genes in hairy roots overexpressing *VrNAC25*. Values are means ± standard deviation (SD). Error bars represent the standard deviation (*n* = 3). Statistical significance determined using Student’s *t*-test (*p* < 0.05).

To confirm the involvement of VrNAC25 in anthocyanin biosynthesis in mung bean sprouts, the function of VrNAC25 was further investigated using a transient expression system in mung bean hairy roots. Hairy roots overexpressing VrNAC25 (OE-NAC25#1 and #2) developed pronounced purple pigmentation on the epidermis (Figure 3A), and the anthocyanin content in these transgenic hairy roots was significantly higher than that in the empty vector control (Figure 3B). RT-qPCR analysis showed that the expression level of *VrNAC25* in overexpressing lines was significantly higher than that in the control, indicating successful expression of the introduced *VrNAC25* gene in mung bean hairy roots. VrMYB90, an R2R3-MYB transcription factor previously confirmed by our lab to positively regulate anthocyanin biosynthesis [15], also showed significantly upregulated expression in VrNAC25-overexpressing hairy roots. Furthermore, transcripts of key anthocyanin biosynthetic genes (*VrCHS*, *VrDFR*, *VrLDOX*) and the transporter *VrGSTF11* were markedly upregulated in VrNAC25-overexpressing hairy roots. (Figure 3C). These results demonstrate that VrNAC25 can positively regulate anthocyanin biosynthesis in mung bean.

### 2.4. VrNAC25 Cannot Directly Bind to the Promoters of VrDFR or VrLDOX

**Figure 4:**
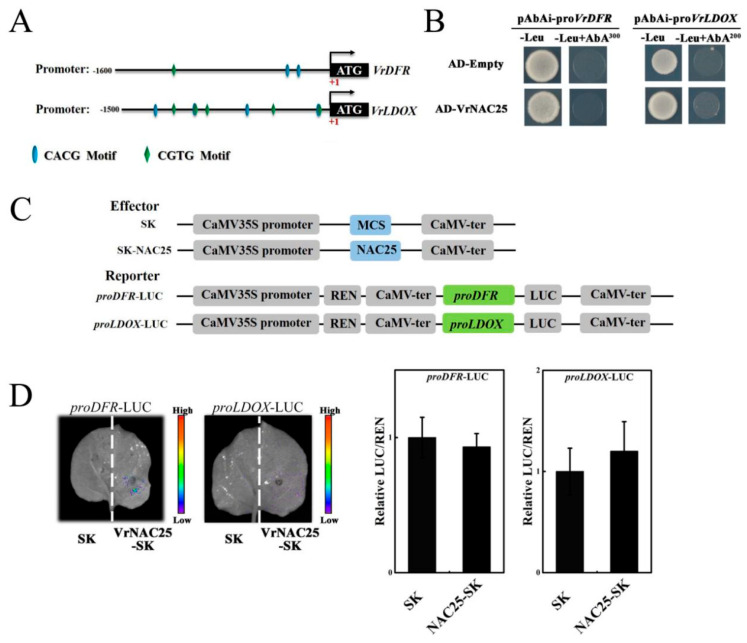
VrNAC25 cannot bind to the promoters of *VrDFR* or *VrLDOX*. (**A**) *Cis*-element analysis in promoter of *VrDFR* and *VrLDOX* genes. (**B**) Detection of VrNAC25 binding to *VrDFR* and *VrLDOX* promoters, respectively, by yeast one-hybrid. (**C**) Schematic diagram of the vector structure used in the dual-luciferase reporter assay system. (**D**) Transcriptional regulation of *VrDFR* and *VrLDOX* by VrNAC25 in tobacco leaves was detected by Plant Living Imaging System. Quantitative analysis (LUC/REN value) of the transcriptional regulation of *VrDFR* and *VrLDOX* by VrNAC25 in tobacco leaves. Values are means ± standard deviation (SD). Error bars represent the standard deviation (*n* = 3).

In VrNAC25-overexpressing mung bean hairy roots, the expression levels of anthocyanin biosynthetic structural genes (*VrCHS*, *VrDFR*, and *VrLDOX*) and the transporter gene *VrGSTF11* were significantly upregulated. We thus hypothesized that VrNAC25 might directly bind to the promoters of *VrDFR* and *VrLDOX* to regulate their expression. Promoter sequences of these two genes were cloned, and cis-acting element prediction using the online tool PlantCARE (https://bioinformatics.psb.ugent.be/webtools/plantcare/html/, accessed on 27 November 2025) revealed the presence of NAC-binding elements (CACG/CGTG) in these two promoters, although with varying numbers (Figure 4A).

Yeast one-hybrid assays were conducted to test potential direct binding of VrNAC25 to the *VrDFR* and *VrLDOX* promoters. Yeast colonies co-transformed with AD-VrNAC25 and pAbAi-pro*VrDFR*/*LDOX* failed to grow on AbA-supplemented media (Figure 4B), indicating the absence of direct binding.

A tobacco leaf dual-luciferase reporter assay was further performed to validate the yeast one-hybrid results. The CDS sequence of VrNAC25 was cloned into the pGreenⅡ-62SK vector as the effector, and the promoters of *VrDFR* and *VrLDOX* were cloned into the pGreenⅡ-0800-Luc vector as reporters. All constructs were transformed into *A. tumefaciens* strain GV3101. REN (Renilla luciferase) was used as an internal reference, and tobacco leaf regions coinfiltrated with the empty SK vector and the corresponding promoter-driven Luc vector served as controls (Figure 4C). Bioluminescence imaging revealed no detectable LUC signal in tobacco leaves co-infiltrated with VrNAC25-SK and *proDFR*-Luc or *proLDOX*-Luc constructs, consistent with the control. Quantitative analysis also showed no significant difference in LUC/REN ratios between the co-infiltrated groups and the SK control group (Figure 4D). Yeast one-hybrid and dual-luciferase assays confirmed that VrNAC25 alone cannot directly activate the promoters of anthocyanin structural genes, indicating that it acts indirectly. VrNAC25 likely functions by interacting with other transcription factors to regulate the transcription of anthocyanin biosynthetic genes.

### 2.5. VrNAC25 Interacted with VrMYB90 at Both Protein Level and Transcription Level

**Figure 5:**
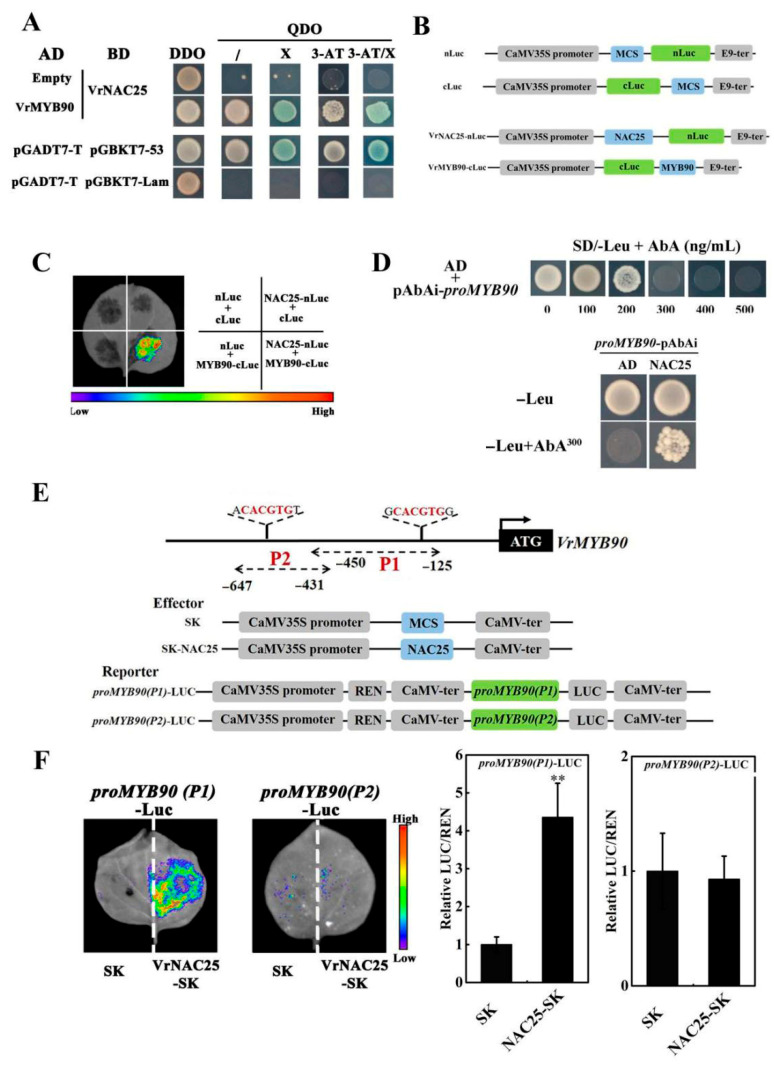
VrNAC25 interacted with VrMYB90 at both protein level and transcription level. (**A**) Detection of the interaction of VrNAC25 with VrMYB90 by yeast two-hybrid. (**B**) Vector structure used in the Luciferase Complementation Assay. (**C**) Detection of the interaction of VrNAC25 with VrMYB90 by Luciferase Complementation Assay. (**D**) Detection of VrNAC25 binding to *VrMYB90* promoters by yeast one-hybrid. (**E**) Schematic diagrams of the promoter structure of *VrMYB90* gene and the vector structure used in the dual luciferase reporter assay system. (**F**) Binding ability of VrNAC25 to different fragments of *VrMYB90* promoter in tobacco leaves was detected by Plant Living Imaging System. Quantitative analysis (LUC/REN value) of the binding ability of VrNAC25 to different fragments of *VrMYB90* promoter in tobacco leaves. The LUC/REN value in tobacco leaves transformed with empty vector (SK) combined with corresponding gene promoter was set to 1. Values are means ± standard deviation (SD). Error bars represent the standard deviation (*n* = 3). Statistical significance determined using Student’s *t*-test (** *p* < 0.01).

We demonstrated that VrNAC25 physically interacts with VrMYB90, and their cooperative action enhances anthocyanin accumulation in mung bean sprouts. To investigate whether VrNAC25 interacts with the regulatory protein VrMYB90, a yeast two-hybrid assay was performed. Yeast two-hybrid assays demonstrated that VrNAC25 interacts with VrMYB90; cotransformed yeast grew on quadruple-deficient medium and exhibited β-galactosidase activity, similar to the positive control (pGADT7-T + pGBKT7-53). In contrast, yeast cells co-transformed with AD and BD-VrNAC25 only grew on DDO media but not on QDO or QDO + 3-AT media, consistent with the negative control (pGADT7-T + pGBKT7-Lam) (Figure 5A). These results indicate that VrNAC25 and VrMYB90 interact in yeast cells.

To further verify their interaction in plant cells, the CDS of VrMYB90 was cloned into the pCAMBIA1300-cLuc vector, and the CDS of VrNAC25 was cloned into the pCAMBIA1300-nLuc vector (Figure 5B). A luciferase complementation assay was performed on tobacco leaves (Figure 5C), and strong fluorescence signal was observed in leaf regions co-infiltrated with NAC25-nLuc and MYB90-cLuc, while no fluorescence was detected in other treatments. These results confirm that VrNAC25 and VrMYB90 interact in plant cells.

To determine whether VrNAC25 directly regulates *VrMYB90* expression, promoter-binding assays were performed. Yeast one-hybrid assays showed that yeast cells co-transformed with AD-VrNAC25 + pAbAi-*proMYB90* grew on both SD/-Leu and SD/-Leu + AbA media, whereas those co-transformed with AD + pAbAi-*proMYB90* did not (Figure 5D), indicating that VrNAC25 can bind to the *VrMYB90* promoter. Based on PlantCARE predictions, the *VrMYB90* promoter was divided into two fragments: P1 (−125~−450 bp) and P2 (−431~−647 bp), which were separately cloned into the pGreenⅡ-0800-Luc vector (Figure 5E) for dual-luciferase reporter assays (Figure 5F). Obvious Luc fluorescence was detected in tobacco leaf regions co-infiltrated with VrNAC25-SK + proMYB90(P1)-Luc, while no significant fluorescence was observed in regions coinfiltrated with VrNAC25-SK + proMYB90(P2)-Luc, which was consistent with the control group. Quantitative analysis (Figure 5F) confirmed these observations. These results demonstrate that VrNAC25 binds to the P1 fragment (−125~−450 bp) of the VrMYB90 promoter to activate its expression, rather than the P2 fragment (−431~−647 bp).

### 2.6. The Interaction Between VrNAC25 and VrMYB90 Further Enhances the Positive Regulation of VrDFR and VrLDOX

**Figure 6:**
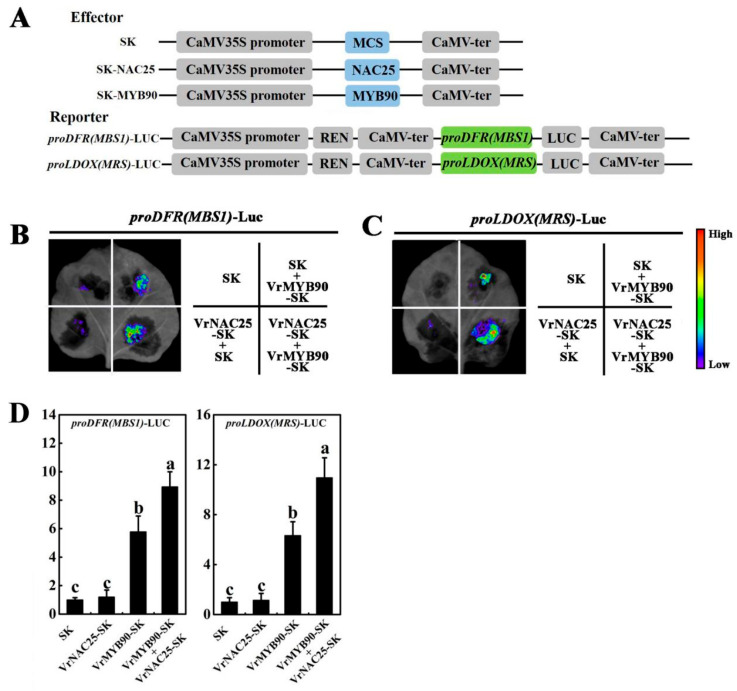
The effect of interaction between VrNAC25 and VrMYB90 on the regulation of anthocyanin synthesis genes. (**A**) Vector structure used in the dual-luciferase reporter assay system; (**B**,**C**) The effect of VrNAC25 interaction with VrMYB90 on VrMYB90 binding to *VrDFR* (**B**) and *VrLDOX* (**C**) promoters in tobacco leaves was detected by Plant Living Imaging System; (**D**) Quantitative analysis (LUC/REN value) of the interaction of VrNAC25 with VrMYB90 in tobacco leaves on the regulation of *VrDFR* and *VrLDOX* gene expression by VrMYB90. The LUC/REN value in tobacco leaves transformed with empty vector (SK) combined with corresponding gene promoter was set to 1. Values are means ± standard deviation (SD). Error bars represent the standard deviation (*n* = 3). Statistical significance determined using Student’s *t*-test (*p* < 0.05).

The above experiments confirmed that VrNAC25 interacted with VrMYB90 to further promote anthocyanin biosynthesis. We thus hypothesized that the interaction between VrNAC25 and VrMYB90 might enhanced the regulatory effect of VrMYB90 on *VrDFR* and *VrLDOX*. A dual-luciferase reporter assay was performed on tobacco leaves with vector constructs as shown in Figure 6A. Plant living imaging showed that in leaves infiltrated with proVrDFR(MBS1)-Luc (Figure 6B), obvious Luc fluorescence was detected in regions containing only VrMYB90-SK, while no significant fluorescence was observed in regions containing only VrNAC25-SK (consistent with the control). However, co-expression of VrNAC25 and VrMYB90 resulted in a significantly stronger LUC signal from *proVrDFR*(MBS1)-Luc and *proVrLDOX*(MRS)-Luc reporters compared with VrMYB90 alone, indicating synergistic activation (Figure 6C). Quantitative analysis confirmed that the LUC/REN ratios in regions co-infiltrated with VrNAC25-SK + VrMYB90-SK + *proVrDFR* (MBS1)-Luc and VrNAC25-SK + VrMYB90-SK + *proLDOX* (MRS)-Luc were significantly higher than those in the control and the group infiltrated with VrMYB90-SK alone. These results indicate that the interaction between VrNAC25 and VrMYB90 further enhances the positive regulation of *VrDFR* and *VrLDOX* by VrMYB90.

## 3. Discussion

Numerous studies have demonstrated that NAC transcription factors play important regulatory roles in anthocyanin biosynthesis [8,16,17]. NAC-type transcription factors regulating anthocyanin biosynthesis have been identified in species such as *Arabidopsis thaliana* and apple, but not yet in mung bean. Genes with high homology often exhibit similar functions. In this study, a phylogenetic tree of VrNAC25 and NAC family genes from *A. thaliana* was constructed, revealing that VrNAC25 clusters with ANAC025 and shares the highest homology (Figure 1A). Although the role of ANAC025 in regulating anthocyanin biosynthesis has not been reported, previous studies showed that during early seed germination in *A. thaliana*, the expression levels of genes critical for anthocyanin biosynthesis (e.g., *MYB90*, *PAL2*, and *GSTF12*) are significantly higher in wild-type plants than in *nac25* mutants [18]. ANAC025 might play a positive regulatory role on regulating anthocyanin biosynthesis, but the specific function need to be solved in the further work. Based on these, we further speculated that VrNAC25, which shares extremely high homology with ANAC025 in mung bean, may also play an important role in regulating anthocyanin biosynthesis. In this study, *VrNAC25* expression was significantly upregulated under light exposure, suggesting that VrNAC25 may mediate light-induced anthocyanin biosynthesis.

To explore the specific regulatory mechanism of VrNAC25 in anthocyanin biosynthesis, detailed experiments were conducted. Subcellular localization showed that VrNAC25 is localized in the nucleus and has transcriptional activity, suggesting that it may function as a nuclear factor in regulatory processes. Using a transient expression system in mung bean hairy roots, anthocyanin content and the expression of related genes were significantly higher in VrNAC25-overexpressing hairy roots than in the control. Notably, anthocyanins accumulated locally in transgenic hairy roots, indicating that VrNAC25, like MYB-type transcription factors, also play a fine-tuning role in mung bean anthocyanin biosynthesis, similar to the function of SlAN2-like in tomato [19].

Reports have shown that NAC transcription factors can regulate anthocyanin biosynthesis by binding to the promoters of structural genes. For example, LcNAC13 interacts with the promoters of *LcCHS1*, *LcCHS2*, and *LcDFR* to negatively regulate anthocyanin biosynthesis in litchi [20]. In our study, the expression levels of late-stage genes *DFR* and *LDOX* were significantly upregulated in transgenic hairy roots (Figure 3C). We thus predicted the cis-acting elements in the promoters of *VrDFR* and *VrLDOX* to investigate whether VrNAC25 also acts by binding to structural gene promoters. Both promoters contained core NAC-binding elements (CACG or CGTG), suggesting potential interaction and regulatory effects. However, yeast one-hybrid and tobacco dual-luciferase reporter assays showed that VrNAC25 cannot directly bind to the promoters of *VrDFR* and *VrLDOX*. Although these two genes have predicted binding sites, they cannot be bound by VrNAC25 and their expression cannot be regulated in the tobacco system. We speculated that there might be two reasons as follows: (1) The binding ability of VrNAC25 to these two genes was validated in vitro in the yeast system, but the actual binding results in vivo could be validated through ChIP-qPCR in the future work. (2) Similarly, in vitro validation of the regulatory ability of VrNAC25 on these two genes in the tobacco system may not necessarily reflect its true regulatory ability in vivo. Subsequently, VrNAC25 related genetic transformation materials can be constructed to determine whether VrNAC25 has a regulatory effect on these two genes by measuring their expression levels in the materials.

In contrast, the VrMYB90 promoter contains core NAC-binding elements (CACG or CGTG), and yeast one-hybrid and tobacco dual-luciferase reporter assays confirmed that VrNAC25 binds to the *VrMYB90* promoter (−125~−450 bp) and positively regulates its expression, consistent with previous reports [14]. Yeast two-hybrid and luciferase complementation assays further confirmed the interaction between VrNAC25 and VrMYB90 in mung bean. To clarify the mechanism by which the VrNAC25-VrMYB90 interaction regulates anthocyanin biosynthesis, tobacco leaf dual-luciferase reporter assays showed that this interaction enhances the transcriptional activation of *VrDFR* and *VrLDOX* by VrMYB90, thereby promoting anthocyanin accumulation. This is consistent with findings in potato [21] and apple [17]. MYB family transcription factors can not only regulate anthocyanin synthesis independently, but also form complexes with other family transcription factors to jointly regulate anthocyanin synthesis through protein–protein interaction [22]. MYB usually forms MBW complexes with bHLH and WD-40 family transcription factors, participating in the regulation of anthocyanin synthesis [23,24,25]. In recent years, MYB has also been reported to co-regulate or antagonize the synthesis of anthocyanins with NAC family transcription factors [8,26]. VrNAC25 can interact with VrMYB90, and their synergistic effects on anthocyanin synthesis suggest that they may form a complex in regulating anthocyanin.

Mung bean sprouts, as one of the most important and widely consumed sprouts in the East Asian market, have high nutritional value and abundant bioactive substances. As one of the largest transcription factor families in plants, NAC family genes play an important role in plant growth and development, stress resistance, and regulation of secondary metabolite synthesis. However, the mechanism by which NAC family transcription factors regulate anthocyanin synthesis in mung beans has not been reported. Therefore, the relevant conclusions of this article clarify the mechanism of the NAC family gene *VrNAC25* in regulating anthocyanin biosynthesis in mung bean sprouts, providing materials and scientific basis for the production of mung bean sprouts rich in anthocyanins. These findings establish a foundation for future studies on the molecular regulation of anthocyanin biosynthesis and may facilitate the development of mung bean cultivars with improved pigmentation and nutritional value. However, for mung beans themselves, the increase in anthocyanins also holds significant importance. After seed germination, seedlings quickly undergo photomorphogenesis upon receiving light signals [27]. The length of the hypocotyl of seedlings will be significantly inhibited, chlorophyll synthesis in cotyledons will rapidly increase, and the content of flavonoids, including anthocyanins, will also increase [28]. Synthesizing more anthocyanins may help seedlings resist excessive growth inhibition caused by high light intensity and other stresses during their early years, considering that anthocyanins are a type of endogenous strong antioxidant in plants [29,30]. Therefore, in this study, VrNAC25 can respond to light signals to promote the synthesis of anthocyanins in the hypocotyls of mung bean sprouts, providing a theoretical basis for enriching the regulatory network of mung bean photomorphogenesis. However, more detailed mechanisms still need to be further analyzed, such as how light signals are transmitted to VrNAC25. Whether HY5 or PIFs in mung bean can regulate the expression of *VrNAC25?* Any other light signaling component such as COP1 could affect VrNAC25 function through protein–protein interaction? All these need to be solved in our further work.

In our research, there are still some issues that need to be addressed in subsequent experiments, mainly including: (1) constructing stable transgenic mung bean lines overexpressing *VrNAC25*, and further screening downstream regulatory genes of VrNAC25 and identifying interaction sites using ChIP-seq/qPCR through stable these transgenic materials. It also could solve the question that whether there are specific cis-elements for NAC binding on condition of variability across promoter subregions. (2) How different light-related processing conditions such as light quality and circadian rhythm affect the synthesis of mung bean anthocyanin through VrNAC25 also needs further exploration in the future.

## 4. Materials and Methods

### 4.1. Experimental Materials and Cultivation Conditions

Mung bean varieties SuLv1and M0313 were provided by the Prof. Li Xin, College of Life Sciences, Nanjing Agricultural University. Seeds were sterilized with 1% sodium hypochlorite for 10 min, rinsed thoroughly, and germinated on moist filter paper at 25 °C in darkness for 48 h. Seedlings were then transferred to a growth chamber at 25 °C with 70% relative humidity under continuous white light (200 μmol m^−2^ s^−1^) for light treatment, or maintained in darkness as controls. The water volumn for each plant was 150 mL, and the medium was renewed every 3 days. At least 3 samples were selected for every treatment as biological replicates.

### 4.2. Determination of Anthocyanin Content

The determination of anthocyanin content was based on previous methods with slight modifications [15]. The hypocotyl of mung bean sprouts were cut into pieces and mixed well. 0.5 g of the sample was then soaked in 5 mL of 1% HCl-methanol solution at room temperature in the dark for 24 h, then extracted with ultrasound for 1 h. After centrifugation at 10,000 rpm for 10 min at room temperature, the absorbance of supernatant solution was measured at 530 nm and 657 nm wavelengths using a UV spectrophotometer (Yoke, Shanghai, China). The content of anthocyanins was calculated according to the following formula: (A530 − 0.25 × A657)/FW.

### 4.3. Extraction of Total RNA, DNA Digestion, and RNA Reverse Transcription

Total RNA was isolated using the Plant RNA Extraction Kit (Takara, Kyoto, Japan) according to the manufacturer’s protocol. cDNA was synthesized using the PrimeScript RT reagent kit (Takara) and used as a template for qRT–PCR. VrActin served as the internal reference gene for normalization. Relative expression levels were calculated using the 2^−ΔΔCt^ method as mentioned in our previous study [15]. The VrActin primer sequnces were as follows: F: TTGCTGGTGATGATGCTCCAAGGGC; R: TTTGCCCCATCCCAACCATCACACC.

### 4.4. Protein Sequence Alignment and Phylogenetic Tree Construction of VrNAC25

Use DNAMAN software (version 9.0.1.116) to predict and translate the protein sequence of VrNAC25 gene. After downloading the protein sequences of all NAC transcription factor families in *Arabidopsis* and NAC transcription factor protein sequences reported to be involved in anthocyanin synthesis in other plants from the NCBI database. Phylogenetic analysis was constructed using MEGA X (version 12.1) with neighbor-joining (NJ) criteria and verified using the maximum likelihood (ML) method. The 1000 bootstrap replicates were performed based on the multiple alignments of the protein sequences encoded by *NAC* genes. NJ analysis was performed using the protein Poisson distances and the pairwise deletion of gap sites. The default parameters were used for ML analysis.

### 4.5. Subcellular Localization of VrNAC25 in Tobacco Cell

For subcellular localization, the VrNAC25 open reading frame (ORF) was cloned into pCAMBIA1300-GFP under the CaMV 35S promoter. *Agrobacterium tumefaciens* GV3101 carrying the construct was infiltrated into *Nicotiana tabacum* leaves at OD_600_ = 0.8. After 8 h incubation in darkness and then 3 days in photoperiod (16 h light/8 h dark, 25 °C, 60% humidity), GFP fluorescence was observed under a Leica TCS SP8 confocal microscope (excitation = 488 nm; emission = 505–530 nm). Empty GFP was used as a control, and the nuclear marker was PIP2A-RFP.

### 4.6. Transcriptional Activation Activity Analysis of VrNAC25

The successfully constructed pGBKT7 (BD)—VrNAC25 vector was transformed into AH109 yeast using the heat shock method and then incubated in a constant temperature incubator at 30 °C for 3–4 days. If the colony can grow on the SD/-Trp/-His/-Ade plate and turn blue on the SD/-Trp/-His/-Ade/-X-α-Gal plate, it indicates that the transcription factor has transcriptional activation activity. Otherwise, it does not (pGADT7-T+pGBKT7-53 is the positive control, pGADT7-T+pGBKT7 Lam is the negative control).

### 4.7. Yeast One-Hybrid Experiment

For yeast one-hybrid assays, *VrDFR* and *VrLDOX* promoters were inserted into pAbAi and co-transformed with pGADT7–VrNAC25 into Y1HGold; Aureobasidin A (200 ng mL^−1^) was used for selection.

### 4.8. Yeast Two-Hybrid Experiment

Yeast two-hybrid assays were conducted using the GAL4 system (Clontech, Mountain View, CA, USA). The VrNAC25 CDS was inserted into pGBKT7 (bait) and VrMYB90 into pGADT7 (prey). *Saccharomyces cerevisiae* strain Y2H Gold was transformed following the manufacturer’s instructions. Transformants were first selected on SD/–Leu/–Trp and subsequently verified on SD/–Leu/–Trp/–His/–Ade with X-α-Gal.

### 4.9. Dual Luciferase Assay

Dual-luciferase reporter assays were conducted in *Nicotiana benthamiana* leaves to analyze promoter activation. Promoters of *VrDFR*, *VrLDOX*, and *VrMYB90* were cloned into the pGreenII 0800-LUC vector kept in lab. Effector constructs carrying VrNAC25 and VrMYB90 in pCAMBIA1300 were co-infiltrated into *A. tumefaciens* GV3101 cultures at OD_600_ = 0.6 (1:1 ratio). After 48 h incubation at 25 °C, luciferase activity was measured using the Dual-Luciferase^®^ Reporter Assay System and quantified with a GloMax 96 Microplate Luminometer(Promega, Madison, WI, USA). LUC/REN ratios were used to determine promoter activation.

### 4.10. Transient Expression Assays in Mung Bean Hairy Roots

Mung bean seeds were sterilized using vacuum chlorine gas for 3–4 h. Germinated seeds on GM medium were subjected to *A. rhizogenes* K599 infection with the pFG5941-VrNAC25 vector for 30 min. Explants were transferred to white solid medium for 5 days in the dark and approximately 4 weeks under light/dark conditions.

### 4.11. Data Analysis

All experiments were performed in triplicate. Data were analyzed using SPSS v26.0 (IBM, Armonk, NY, USA). Statistical significance was assessed using Student’s *t*-test or one-way ANOVA followed by Tukey’s post hoc test (*p* < 0.05). Values are presented as mean ± SD.

## 5. Conclusions

In conclusion, VrNAC25 functions as a positive regulator of anthocyanin biosynthesis in mung bean sprouts by interacting synergistically with VrMYB90. VrNAC25 does not directly bind to the promoters of structural anthocyanin genes but indirectly activates their transcription through cooperation with VrMYB90. These findings enhance our understanding of the transcriptional regulation of anthocyanin biosynthesis and provide a theoretical foundation for improving the nutritional and economic value of mung bean sprouts.

## Data Availability

Data are contained within the article.

## Supplementary Materials

The following supporting information can be downloaded at: https://www.mdpi.com/article/10.3390/plants14233667/s1, Figure S1: The expression level of VrNAC25 in transcriptome conducted between M0313 and SuLv 1. The expression level was calculated with FPKM (Fragments Per Kilobase of exon model per Million mapped fragments) value.
