# Supplementary figures and images for: VrNAC25 Promotes Anthocyanin Synthesis in Mung Bean Sprouts Synergistically with VrMYB90
Source: Plants (Basel). 2025 Dec 2;14(23):3667. doi: 10.3390/plants14233667 (PMC12693972; doi:10.3390/plants14233667)

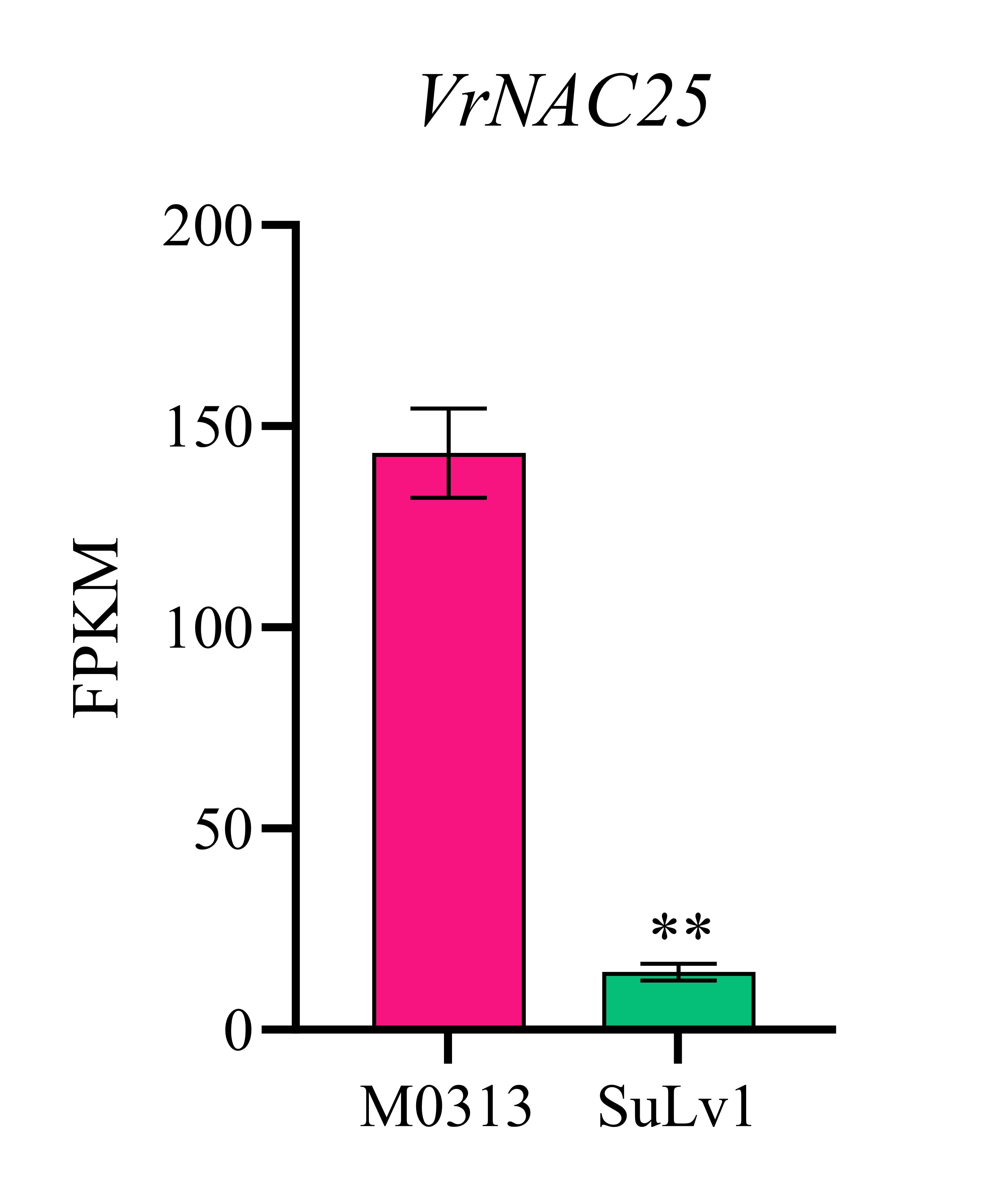

Supplement: Supplementary file 1 [file plants-14-03667-s001.zip › plants-3950650-supplementary.jpg]
